# Lack of a Retinal Phenotype in a Syne-2/Nesprin-2 Knockout Mouse Model

**DOI:** 10.3390/cells8101238

**Published:** 2019-10-11

**Authors:** Nathalie Falk, Anneka Joachimsthaler, Kristin Kessler, Uwe Thorsten Lux, Angelika Anna Noegel, Jan Kremers, Johann Helmut Brandstätter, Andreas Gießl

**Affiliations:** 1 Animal Physiology, Friedrich-Alexander-Universität Erlangen-Nürnberg, 91058 Erlangen, Germany; nathalie.falk@ukr.de (N.F.); Anneka.Joachimsthaler@uk-erlangen.de (A.J.); kristin.kessler@uni-wuerzburg.de (K.K.); uwe.lux@fau.de (U.T.L.); johann.helmut.brandstaetter@fau.de (J.H.B.); 2 Department of Ophthalmology, University Hospital Erlangen, 91054 Erlangen, Germany; Jan.Kremers@uk-erlangen.de; 3 Institute of Biochemistry I; Medical Faculty, University Hospital, University of Cologne, 50931 Cologne, Germany; noegel@uni-koeln.de

**Keywords:** Syne-2, Pericentrin, retina, nuclear migration, primary cilium, ciliogenesis

## Abstract

Syne-2 (also known as Nesprin-2) is a member of a family of proteins that are found primarily in the outer nuclear membrane, as well as other subcellular compartments. Syne-2 contains a C-terminal KASH transmembrane domain and is part of a protein network that associates the nuclear envelope to the cytoskeleton via the binding to actin filaments. Syne-2 plays a role in nuclear migration, nuclear positioning during retinal development, and in ciliogenesis. In a previous study, we showed a connection between Syne-2 and the multifunctional scaffold protein Pericentrin (Pcnt). The elimination of the interaction of Syne-2 and Pcnt showed defects in nuclear migration and the formation of outer segments during retinal development, as well as disturbances in centrosomal migration at the beginning of ciliogenesis in general. In this study, the Syne-2 KO mouse model Nesprin-2△ABD (Syne-2^tm1Ngl^, MGI) with special attention to Pcnt and ciliogenesis was analyzed. We show reduced expression of Syne-2 in the retina of the Syne-2 KO mouse but found no significant structural—and only a minor functional—phenotype. For the first time, detailed expression analyses showed an expression of a Syne-2 protein larger than 400 kDa (~750 kDa) in the Syne-2/Nesprin-2 KO mouse. In conclusion, the lack of an overt phenotype in Syne-2/Nesprin-2 KO mice suggests the usage of alternative translational start sites, producing Syne-2 splice variants with an intact Pcnt interaction site. Nevertheless, deletion of the actin-binding site in the Syne-2/Nesprin-2 KO mouse revealed a high variability in scotopic oscillatory potentials assuming a novel function of Syne-2 in synchronizing inner retinal processes.

## 1. Introduction

Syne-2 (Nesprin-2, NM_001005510.2; ENSMUST00000044217) is a nuclear envelope protein that plays a role in nuclear migration, nuclear positioning during retinal development [1,2,3], and in ciliogenesis [4]. Recently, we identified Syne-2/Nesprin-2 as a novel interaction partner of Pericentrin (Pcnt) [5], which is a component of centrosomes and cilia, where it serves as a multifunctional scaffold for anchoring numerous proteins and protein complexes and thus is involved in many biological processes, e.g., ciliogenesis [6,7,8,9,10,11].

We demonstrated that a down-regulation or knock-out (KO) of Pcnt and Syne-2/Nesprin-2 causes defects in nuclear migration and ciliogenesis in cell culture and in developing photoreceptors [5]. Furthermore, we provided evidence that the interaction of Pcnt with Syne-2/Nesprin-2 is required for ciliogenesis in differentiated cells, as well as for the attachment of the centrosome to the nucleus in cycling cells, which is crucial for correct nuclear migration and cell polarization.

In an effort to achieve a better functional characterization of the Syne-2/Nesprin-2 interaction in the retina, we analyzed in the present study the only available Syne-2 KO mouse model Nesprin-2△ABD (Syne-2^tm1Ngl^, MGI). This mouse lacks the actin-binding domain (ABD) at the N-terminus of Syne-2 (exons 2-4). Previous characterization of the mouse model revealed reduced expression levels of smaller splice variants and the complete absence of variants with a molecular weight larger than 400 kDa (full-length protein 796 kDa) [12]. Based on these results, we assumed that the deletion would also affect the structure of the remaining protein and, therefore, the Pcnt/Syne-2 binding site at the N-terminus of the Syne-2/Nesprin-2 protein [5]. In the Syne-2/Nesprin-2 KO mouse retina, Syne-2 expression was attenuated in photoreceptors but Syne-2 protein localization was not altered. Furthermore, Pcnt localization and photoreceptor integrity in the retina of Syne-2/Nesprin-2 KO mice were comparable to wild-type (WT) controls and ERG recordings of Syne-2/Nesprin-2 KO retinae did not reveal an altered photoreceptor function. For the first time, detailed expression analyses finally showed an expression of a Syne-2/Nesprin-2 protein with a molecular weight of larger than 400 kDa in the Syne-2/Nesprin-2 KO mouse. These high molecular weight splice variants showed only a slight reduction of Syne-2/Nesprin-2 protein expression and still contained the Pcnt binding site.

In conclusion, the lack of an overt phenotype in Syne-2/Nesprin-2 KO mice suggests the usage of alternative translational start sites, producing Syne-2 splice variants with an intact Pcnt interaction site.

## 2. Materials and Methods

### 2.1. Ethics Statement

The experiments were performed in compliance with the guidelines for the welfare of experimental animals issued by the Federal Government of Germany and the University of Erlangen-Nuremberg. The animal experiments were approved and registered by the local authorities (Regierung von Mittelfranken, AZ54-2531.31-26/07; Amt für Veterinärwesen der Stadt Erlangen, AZ TS-10/07 Lehrstuhl für Zoologie-Tierphysiologie). Mouse breeding was performed in the animal facilities of the University of Erlangen-Nuremberg according to European and German laws on experimental animal welfare (Tierschutzgesetz; AZ820-8791.2.63).

### 2.2. Animals

Adult C57BL/6JRj and Nesprin-2∆ABD (Syne-2^tm1Ngl^, MGI) [12] mice (age 2–4 months) of either sex were used in this study. In all experiments, littermates of the mouse strain Nesprin-2∆ABD (Syne-2^tm1Ngl^, MGI) with the different genotypes (+/+ = WT, +/− = heterozygote, −/− = KO) were used. The animals were kept in a 12 h/12 h light/dark cycle with lights on at 6:00 A.M. and with food and water ad libitum. Information about the original generation of the Nesprin-2∆ABD (Syne-2^tm1Ngl^, MGI) mouse line is available in [12].

### 2.3. Western Blot Analysis

Western blot analysis was performed as described by [10]. Briefly, fresh isolated tissues were homogenized in extraction lysis buffer (50 mM Tris-HCl (pH 7.5), 150 mM NaCl, 1% Triton X-100, 0.5% Natrium-Deoxycholat) and fresh proteins were separated by SDS-PAGE using 10% polyacrylamide gels, 3–8% NuPAGE Novex Tris-Acetate Gels (Life Technologies, Carlsbad, CA, USA) or 4–15% Mini-PROTEAN TGX Stain-Free (Bio-Rad Laboratories, Munich, Germany). Blotting was performed in a semi-dry blot chamber (Trans-Blot Turbo Transfer System, Bio-Rad Laboratories, Munich, Germany) by 25 V for 30–40 min at RT to polyvinylidene difluoride (PVDF) membranes (Amersham Hybond- P, GE Healthcare or Transblot Turbo Midi-size LF PVDF membranes, Bio-Rad Laboratories). The membrane was blocked 1 h with blocking solution (0.2% Applichem Blocking Reagent (Darmstadt, Germany), 10 mM Tris, 150 mM NaCl, pH 7.4) and probed with primary antibodies (overnight at 4°C) and horse-radish peroxidase-labeled secondary antibodies (described in the section “antibodies”). Imaging and quantification (mean band intensity measurement normalized to total protein with stain-free technique) were performed with a molecular imager, “stain-free technology”, and the software Image Lab (ChemiDoc XRS; Bio-Rad Laboratories). Protein separation can be visualized on the gel after electrophoresis. Protein transfer to the membrane can be confirmed and assessed prior to blot detection and blot normalization can occur without the extra steps required for typical reference protein detection.

### 2.4. Immunofluorescence and Light Microscopy

Preparation of retinal tissue and antibody incubation for immunofluorescence microscopy were done as described previously [10]. Unfixed or 0.4% PFA (10 min at RT) sections were incubated with 0.01% Tween 20 in PBS for 10 min and washed 15 min in PBS. Next, samples were blocked for 45 min in blocking solution (0.5% cold-water fish gelatin and 0.1% ovalbumin in PBS). Primary antibody incubation was carried out overnight in blocking solution at 4 °C. Next day the samples were washed 3 × 10 min in and incubated with secondary antibodies in blocking solution with DAPI (4,6-diamidino-2-phenylindole) (1:50,000; Sigma-Aldrich, Taufkirchen, Germany). After additional 3 × 10 min PBS washing steps, samples were mounted in Aqua Poly Mount (Polysciences, Eppelheim, Germany). For hematoxylin and eosin staining sections were fixed with 4% PFA (5 min at RT) and rinsed with deionized water. The sections were then stained with Harris’ hematoxylin solution for 3 min and rinsed in tap water for an additional 3 min. Afterwards, samples were incubated in eosin solution for 2 min, rinsed with deionized water twice, dehydrated with ascending ethanol and mounted in EUKITT Neo (O. Kindler ORSAtec, Bobingen, Germany,). Stained sections were analyzed with a Zeiss Axio Imager Z2 equipped with an ApoTome by using ZEN blue 2012 software or imaged on a Laser Scanning Microscope 710 by using ZEN black 2010 software with corresponding imaging modules (Zeiss, Oberkochen, Germany). Images were adjusted for contrast and brightness using Adobe Photoshop CS6 (Adobe Systems, San Jose, CA, USA) and figures were arranged using Corel Draw X8 (Corel GmbH, Munich, Germany). In order to study three-dimensional (3D) protein distributions, z-stacks were taken (stack interval: 0.193–0.195 μm) and fused to 3D images by Imaris (version 8.1, Oxford Instruments, Abingdon, Oxon UK). For better comparison, fluorescence intensity profiles were generated using FIJI [13]. For each image, twenty vertical line profiles were obtained at regular intervals across the total thickness of the retina. Results were averaged and normalized to the maximum measured distance. Intensity profiles of DAPI staining were used to identify retinal cell layers.

### 2.5. Antibodies

For immunocytochemistry on cryostat sections (ICC) and Western blot analyses (WB), the following antibodies were used: rabbit anti-Pcnt MmPeriC1/C2 pAb (ICC 1:500; WB 1:250) previously described in [10]; mouse anti Syne-2 F-11 mAb (ICC 1:100; WB 1:250; #sc-398616, Figure 2), rabbit anti-PCM1 pAb (ICC 1:700; #sc-67204) (both Santa Cruz Biotechnology, Santa Cruz, CA, USA); mouse Brn-3A (ICC 1:500; #AB5945), rabbit anti-PC2 pAb (ICC 1:100; #AB9088) (both Merck KGaA, Darmstadt, Germany); mouse anti-Piccolo (ICC 1:1000; #142111; Synaptic Systems, Göttingen, Germany); guinea pig VGlutT1 (ICC 1:50000; # AB5905); rabbit PKCα(ICC 1:10000; # SAB1305634) (all Sigma-Aldrich, Merck KGaA, Darmstadt, Germany); polyglutamylated tubulin clone GT335 (ICC 1:1000; # AG-20B-0020-C100) (Biomol, Hamburg, Germany); rabbit anti-ubMunc13-2 (ICC 1:6000) previously described in [14]; mouse anti-Syne-2 K56-386 mAb (ICC 2:1, Figure 2) was a gift from Angelika A. Noegel (Center for Biochemistry, Medical Faculty, University of Cologne, Germany) previously described in [12].

The following secondary antibodies were used: Alexa^TM^ 568 and Alexa^TM^ 488 goat anti-mouse, goat anti-rabbit and goat anti guinea pig IgG conjugates (1:500; Molecular Probes, Eugene, OR, USA); HRP goat anti-mouse/rabbit IgG conjugate (1:10,000; Sigma-Aldrich).

### 2.6. Electron Microscopy

Preparation of the mouse retinae and conventional electron microscopy was done as described previously [5]. For conventional electron microscopy and good tissue preservation, retinae were fixed in 4% PFA and 2.5% glutaraldehyde for 2 h at room temperature. Tissue contrasting was carried out by incubation in 1.5% potassium ferrocyanide and 2% osmium tetroxide in 0.1M cacodylate buffer (pH 7.4) for 1.5 h. Retinae were dehydrated using an ethanol series and propylene oxide with 0.5% uranyl acetate added at the 70% ethanol step. The tissue was embedded in RenLam resin (Serva, Heidelberg, Germany).

### 2.7. RT-PCR

RNA isolation from fresh isolated tissue and RT-PCR analysis were done as described previously [10]. Cycling conditions: 35 cycles at 94 °C for 15 s, 60 °C for 30 s, and 72 °C for 1 min followed by a 10 min 72 °C extension step. Primers specific for mouse Pcnt^917–1169^ (covering all known splice variants) were as follows: forward primer (5′-ATGCTCAGGGCTGACCTGGAGCTGGCTCAG-3′) and reverse primer (5′-CCACTGGACTCTGGTAAGAGATTCCACCTC-3′). Primers specific for Syne-2^1852–2143^ (including the Pcnt interacting site, exon 39-41) were as follows: forward primer (5′-TGTTTTGAACCACCAGAAACAAAA-3′) and reverse primer (5′-TTACTCCTTACTCTGCAAATACTTTTC-3′). Primers specific for Syne-2^1921–2066^ (including the Pcnt interacting site, exon 39-41) were as follows: forward primer (5′-TGCCAGGCCCGAGCAGGGGAACTGAATAAC-3′) and reverse primer (5′-TTATGGCATTTTGCCTTCTGATGAATT-3′). Primers specific for β-Actin were as follows: forward primer (5′-TCACCCACACTGTGCCCATCTACGAG-3′) and reverse primer (5′-ACACAGAGTACTTGCGCTCAGGAGGA-3′). Primers were used for cloning, sequencing, and RT-PCR analysis.

### 2.8. ERG Measurements

Full details of the general setup have been described elsewhere [15]. Briefly, animals (7 wild-type and 7 Nesprin-2∆ABD mice KO) were dark-adapted overnight for electroretinographic (ERG) measurements. All further handling was done under dim red light to maintain dark adaptation. Animals were anesthetized by an intramuscular injection of 50 mg/kg ketamine (Ketavet^®^, Pfizer Deutschland GmbH, Berlin Germany) and 10 mg/kg xylazine (Rompun^®^ 2%, Bayer HealthCare AG, Leverkusen, Germany). A subcutaneous injection of saline (10 mg/kg 0.9%) kept them hydrated. Additional doses of anesthesia (max. 250 µg ketamine, 50 µg xylazine) were administered if necessary. The pupils were dilated with a drop of tropicamide (Mydriaticum Stulln^®^ 5 mg/mL, Pharma Stulln GmbH, Stulln, Germany) and a drop of phenylephrine-hydrochloride (Neosynephrin POS^®^ 5%, Ursapharm, Saarbrücken, Germany). The animal was then placed on a heated platform inside a Ganzfeld stimulator (Roland Consult Q450 SC, Brandenburg, Germany) to prevent hypothermia. Ground and reference needle electrodes were inserted subcutaneously at the tail base and on the forehead, respectively. Contact lens electrodes (Mayo Corporation, Aichi, Japan) filled with Corneregel^®^ (Dr. Mann Pharma, Berlin, Germany) served as active electrodes. Afterward, the mice were allowed 10 min of dark adaptation. Initially, recordings (RetiPort system, Roland Consult, Brandenburg, Germany) to standard short flashes of varying strengths (white LED-flashes, −2.7, −2.2, −1.7, −1.2, −0.7, −0.2, and 0.8 phot log cd.s/m^2^ flash strength, 8–12 averages depending on flash intensity) to assay rod and combined rod-cone driven retinal function [16] were done. The time between flashes of the same flash strength varied between 2 and 20 s and the time between two different flash strengths varied between 30 and 120 s to ensure dark adaptation. Subsequently, the animals were light-adapted to a 25 cd/m^2^ background for at least 5 min before photopic short-flash ERGs (white LED-flashes, −0.2, 0.3, 0.8, 1.0, and 1.2 phot log cd.s/m^2^ flash strength, 20 sweeps/average) and photopic flicker ERGs were recorded to examine cone driven retinal activity [16] (8, 14, 26, and 30 Hz sine-wave modulation at 100% contrast and 60 cd/m^2^ mean luminance; 40 averages to one second episodes were obtained at each condition). After ERG recordings, the animals were transferred to a heating blanket where they were allowed to wake up.

### 2.9. ERG Data Analysis

Major ERG components (scotopic and photopic a- and b-wave, and scotopic oscillatory potentials isolated from the ascending arm of the scotopic b-wave) were analyzed in terms of their peak/trough amplitudes and implicit times as described elsewhere [17]. The implicit time of the 2^nd^ oscillatory potentials peak was defined from the time-of-stimulation to peak. Each animal value of both eyes were averaged, unless the values of the maximal scotopic b-wave amplitude showed more than 10% difference. In this case, only the eye with the larger values was taken into analysis. The values of all animals of the same genotype were averaged for each flash strength.

All statistical analyses on the ERG data were performed with SPSS (V21, IBM Deutschland GmbH, Berlin, Germany). Mean values from wild-type and mutant groups were compared with a Mann–Whitney-U test because the data did not follow Gaussian distributions (according to Shapiro–Wilk tests). The implicit times of the 2nd oscillatory potential peak was additionally tested for variance equality with Levene’s test. An alpha-value of 0.05 was adopted for all comparisons.

## 3. Results

### 3.1. Syne-2/Nesprin-2 KO Mice Show Normal Retinal Morphology

**Figure 1:**
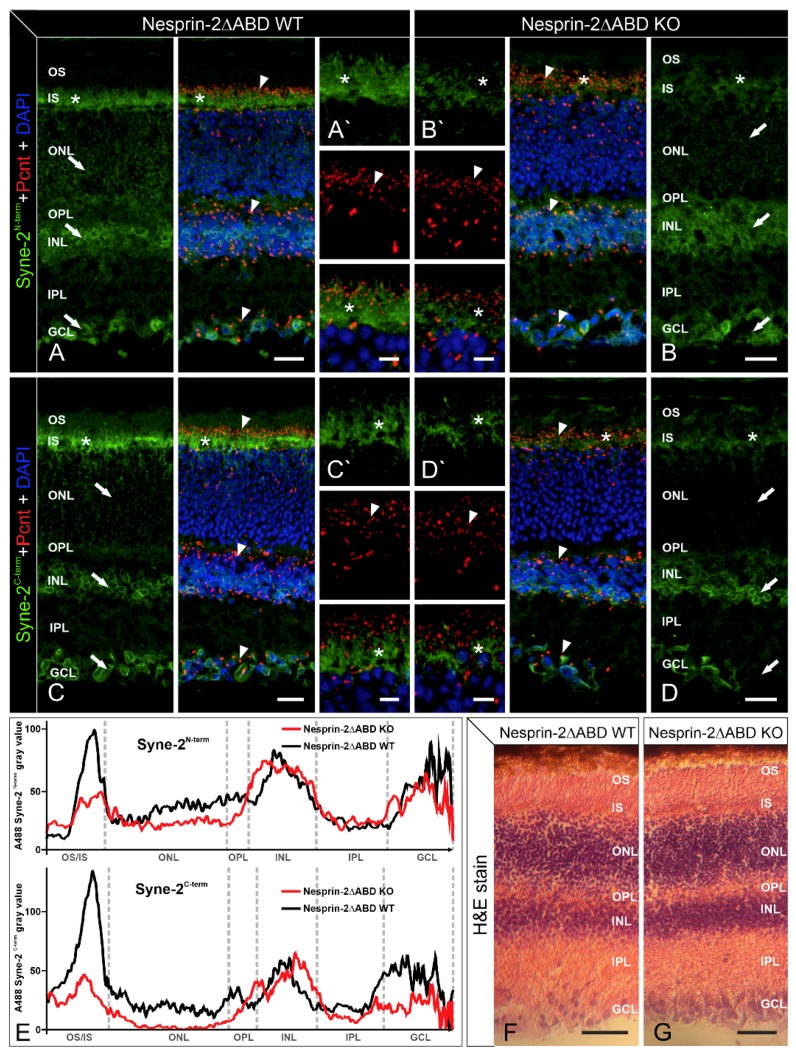
Light microscopic and immunofluorescence analysis of Pcnt and Syne-2 in retinae of Nesprin-2ΔABD mouse strain. Images of vertical sections through adult (age 2–4 month) Nesprin-2ΔABD mouse retinae, stained with antibodies against Syne-2N-term (K56-386, green, A, B) or Syne-2C-term (F-11, green, C, D), Pcnt (MmPeriC1, red), and DAPI (blue). (**A**,**C**) In Nesprin-2ΔABD wild-type (WT) animals, Syne-2 is localized in the region of the inner segments of photoreceptors (asterisks) and the somata of retinal cells in the ONL, INL, and GCL (arrows). Pcnt is present at the ciliary region of photoreceptors and at the centrosomal region of non-photoreceptors (arrowheads). (**B**,**D**) In Nesprin-2ΔABD knockout (KO) animals, Syne-2 staining is only faintly visible in the region of the photoreceptor inner segments (asterisk) and the ONL (arrows). There are no obvious differences between Nesprin-2ΔABD WT and KO retinae with respect to the localization of Pcnt (arrowheads). (**A**–**D**) High-resolution micrographs of the photoreceptor ciliary region. OS: outer segment; IS: inner segment; ONL: outer nuclear layer; OPL: outer plexiform layer; INL: inner nuclear layer; IPL: inner plexiform layer; GCL: ganglion cell layer. (**E**) Mean intensity profiles extracted from images (A–D) show lower Syne-2 fluorescence in the region of the photoreceptor inner segments and the ONL in the Syne-2/Nesprin-2 KO retinae with both antibodies used. (**F**,**G**) Hematoxylin and eosin staining (H&E stain) of Nesprin-2ΔABD WT and KO retinae shows no structural differences. Scale bars: 20 μm (**A**–**D**), 5 µm (**A′**–**D′**), 50 µm (**D**,**G**).

**Figure 2:**
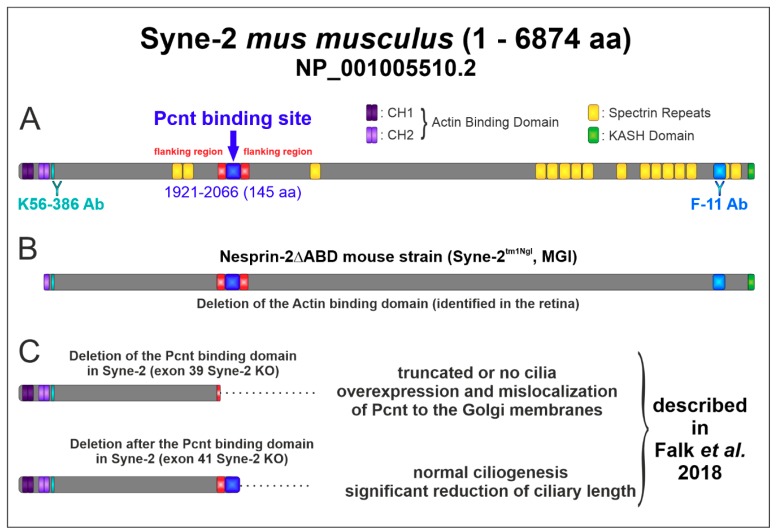
Schematic structure of the Syne-2 protein (NP_001005510.2) and the different deletion forms. (**A**) Structure of Syne-2 containing an N-terminal actin-binding domain composed of two calponin homology domains CH1 and CH2, several spectrin repeats and a C-terminal Klarsicht, ANC-1 and Syne Homology (KASH) domain. The epitopes of anti-Syne-2 antibodies are indicated (K56-386 > N-term domain; F-11 > C-term domain). The localization of the identified Pcnt binding site (exon 39 41, aa residues 1921–2066) is marked in blue [5]. (**B**) Structure of the N-terminal truncated Syne-2 variant identified in the retina of the Nesprin-2∆ABD KO mouse strain. (**C**) Structures of the C-terminal truncated Syne-2 variants in cell culture exhibiting a significant phenotype [5].

In a first step, we analyzed the distribution of Syne-2 and Pcnt in Syne-2/Nesprin-2 KO and WT control retinae with immunocytochemistry and light microscopy (Figure 1). Because of the existence of several Syne-2 splice variants, we used the monoclonal antibodies K56-386 [12] and F11 (Santa Cruz), which recognize epitopes mapping near the N- and C-terminus of Syne-2 (Figure 2).

Both antibodies detected Syne-2 in the Syne-2/Nesprin-2 KO and WT control retinae with Syne-2 immunoreactivity being present throughout the different retinal layers: the photoreceptor inner segments (IS), the outer nuclear membrane of retinal cells in the outer nuclear layer (ONL), the membrane of the inner nuclear (INL), and the ganglion cell layer (GCL). Comparing the staining between the two genotypes, Syne-2 immunofluorescence was noticeably weaker in the IS and the ONL of the Syne-2/Nesprin-2 KO retinae (Figure 1B,D,E) compared to the WT controls (Figure 1A,C,E). Double labeling of Syne-2 and Pcnt showed i) a co-distribution of Syne-2/Pcnt at the ciliary region of the photoreceptors in both genotypes and ii) a Pcnt staining at the centrosomes in the INL and GCL in Nesprin-2△ABD KO retinae that did not differ from WT control retinae (Figure 1A’,B’,C’,D’). Hematoxylin and eosin staining of Nesprin-2ΔABD WT and KO retinae showed no structural differences in the mouse retina (Figure 1F,G). Taken together, we could not detect any significant differences in the overall appearance of the retinal morphology and the expression of Syne-2 and Pcnt in the Syne-2/Nesprin-2 KO mice compared to WT control mice. Only the photoreceptors showed reduced staining of Syne-2 in the Syne-2/Nesprin-2 KO compared to WT control mice (Figure 1E; KO and control mice were littermates in these experiments).

### 3.2. Syne-2/Nesprin-2 KO Mice Show Intact Photoreceptor Connecting Cilia

**Figure 3:**
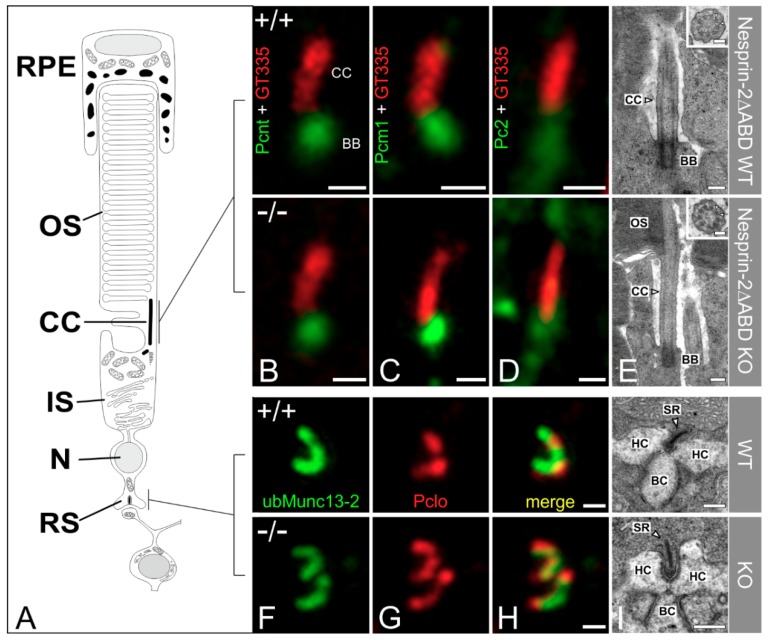
High-resolution microscopical analysis of the basal body complex and the ribbon synapse of Nesprin-2△ABD mouse strain (age 2–4 month). (**A**) Scheme of a vertebrate rod photoreceptor. (**B**–**D**) Photoreceptors connecting cilia (CC) of wild-type and knockout Nesprin-2∆ABD mouse retinae double-labeled with antibodies against Pcnt (B, MmPeriC1, green), Pcm1 (C, green), or Pc2 (D, green) as markers for the basal body complex (BBC) and polyglutamylated tubulin (GT335, red) as a marker for the ciliary apparatus. Pcnt and its interaction partners are localized at the BBC of the connecting cilium of the photoreceptor in wild-type and knockout Nesprin-2∆ABD retinae. (**E**) Representative electron micrographs of rod photoreceptor terminals, processed for best tissue preservation. (**F**–**H**) Presynaptic ribbon complex of wild-type and knockout Nesprin-2∆ABD mouse retinae labeled with antibodies against ubMunc13-2 and Piccolo. (**I**) Representative electron micrographs of rod photoreceptor terminals processed for best tissue preservation. The comparison of wild-type and knockout Nesprin-2∆ABD shows no ultrastructural alterations. Scale bars: 0.5 μm (**B**–**D**), 0.5 μm (**H**), 0.25µm (**E**,**I**), 0.1 μm higher magnification views (**E**).

Next, we asked whether the attenuated Syne-2 immunoreactivity (Figure 1) in the photoreceptors affects the structure of the photoreceptor connecting cilium (CC). We labeled WT control and Syne-2/Nesprin-2 KO retinae with antibodies against Pcnt and the two Pcnt binding proteins polycystin-2 (Pc2) and Pcm1, each in combination with polyglutamylated-tubulin as a marker for the CC, and analyzed the photoreceptor IS with high-resolution confocal laser scanning and electron microscopy (Figure 3A–D).

Pc2 and Pcm1 staining at the CC were indistinguishable between WT control and Syne-2/Nesprin-2 KO retinae (Figure 3B–D). Electron microscopy corroborated the light microscopical findings, showing an intact CC with the typical nine peripheral doublet microtubules in the Syne-2/Nesprin-2 KO retinae (9 × 2 + 0 structure, Figure 2E). In addition, no differences could be detected between Syne-2/Nesprin-2 KO and WT control retinae in the morphology and the layering of the photoreceptor nuclei in the ONL (data not shown). Taken together, the results indicate that the deletion of the N-terminal actin-binding domain in the Syne-2/Nesprin-2 KO mouse does not affect the localization of ciliary proteins or the structure of the photoreceptor CC.

Because we were able to demonstrate that the expression of Syne-2 in photoreceptors in the Syne-2/Nesprin-2 KO is reduced (Figure 1), we also examined the structure of the presynaptic ribbon complex in the photoreceptor terminals in the OPL of Syne-2/Nesprin-2 KO and WT control retinae with light and electron microscopy. Photoreceptor ribbon synapses were stained with antibodies specific for proteins expressed at the synaptic ribbon (Piccolino) and the arciform density compartment (ubMunc13-2) [14,18,19] (Figure 2F–H). We found no differences in the composition and localization of these proteins at the presynaptic ribbon complex in the Syne-2/Nesprin-2 KO compared to WT control retinae. Ultrastructural analysis of the Syne-2/Nesprin-2 KO mouse photoreceptor ribbon synapses showed the typical ribbon synaptic configuration with a presynaptic ribbon facing three to four invaginating postsynaptic elements, horizontal cell processes and bipolar cell dendrites (Figure 3I).

### 3.3. Syne-2/Nesprin-2 KO Mice Show an Intact ERG but Larger Variability in the Timing of the Oscillatory Potentials

**Figure 4:**
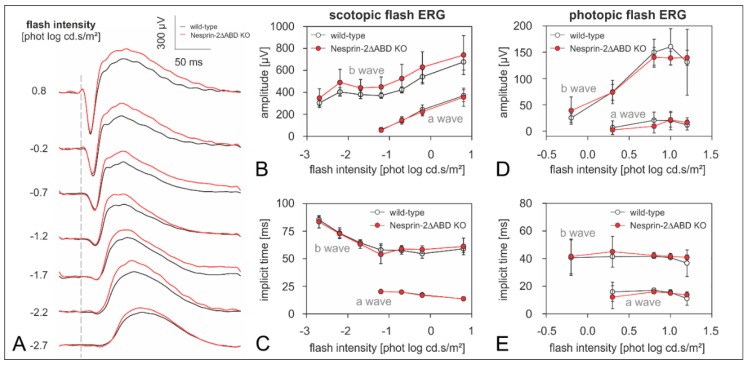
Parameter analysis of scotopic and photopic a- and b-wave in the retina of Nesprin-2ΔABD mouse strain (age 2–4 month). (**A**) ERG responses without oscillatory potentials from wild-type (mean, black curves, *n* = 7) and Nesprin-2∆ABD KO (mean, red curves, *n* = 7) mice evoked with different flash intensities (shown on the left). Dashed line indicates the time point of the stimulus flash. (**B**) Amplitude vs. flash intensity profiles of scotopic a- and b-wave (wild-type: mean ± sd, white symbols, *n* = 7; Nesprin-2∆ABD KO: mean ± sd, red symbols, *n* = 7). (**C**) Implicit time vs. flash intensity profiles of scotopic a- and b-wave (wild-type: mean ± sd, white symbols, *n* = 7; Nesprin-2∆ABD KO: mean ± sd, red symbols, *n* = 7). (**D**) Amplitude vs. flash intensity profiles of photopic a- and b-wave (wild-type: mean ± sd, white symbols, *n* = 7; Nesprin-2∆ABD KO: mean ± sd, red symbols, *n* = 7). (**E**) Implicit time vs. flash intensity profiles of photopic a- and b-wave (wild-type: mean ± sd, white symbols, *n* = 7; Nesprin-2∆ABD KO: mean ± sd, red symbols, *n* = 7). Unpaired t-tests revealed no significant differences between wild-type and Nesprin-2∆ABD KO (all *p* > 0.05).

To examine the physiological integrity of the retina of Nesprin-2△ABD KO mice, we performed full-field ERG measurements on Nesprin-2△ABD KO (*n* = 7) and WT (*n* = 7) mice. We found that both the amplitudes and implicit times of scotopic and photopic flash ERG components recorded from Syne-2/Nesprin-2 KO mice did not differ from those of the WT controls (Figure 4). Although the amplitudes of the Syne-2/Nesprin-2 KO scotopic b-waves were slightly larger than the average WT b-waves for all measured flash strengths (Figure 4A,B), these differences did not reach significance.

**Figure 5:**
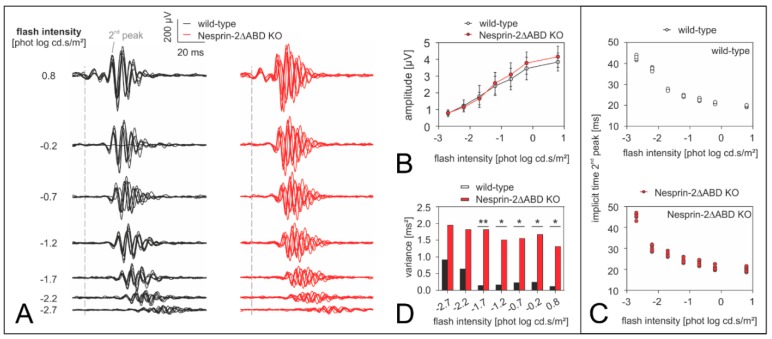
Analysis of the oscillatory potentials isolated from the scotopic flash-ERG in the retina of Syne-2/Nesprin-2 KO mouse strain (age 2–4 month). (**A**) Individual oscillatory potential traces from each wild-type (black curves, *n* = 7) and Syne-2/Nesprin-2 KO mouse (red curves, *n* = 7) are shown for each scotopic flash intensity. Note that the curves were more varied in time (i.e., less synchronized) within the Syne-2/Nesprin-2 KO group. As such, group averaged (mean ± sd) intensity-response amplitudes of the 2nd oscillatory peak are shown in (**B**), whereas individual implicit times plots are displayed in (**C**) so as to better visualize the spread in each group. Although both wild-type and Syne-2/Nesprin-2 KO implicit time profiles exhibited the same trend with increasing flash intensity, the inter-individual variability was larger in the Syne-2/Nesprin-2 KO group. This was more obvious when (**D**) the variances of the implicit times of the two mouse groups were compared. Statistical significance is denoted with a * *p* < 0.05 or ** *p* < 0.005 (Levene’s test).

The mean amplitudes of oscillatory potentials (defined as the maximum amplitude in the frequency domain—i.e., after Fourier analysis of the response—at frequencies above 70 Hz) of WT and Syne-2/Nesprin-2 KO ERGs showed similar dependencies on flash intensity. That is, their mean amplitudes increased and mean implicit times decreased with increasing flash strength (Figure 5A,B).

Interestingly, the oscillatory potentials within the Syne-2/Nesprin-2 KO group (Figure 5A) showed larger interindividual variability than those in the WT group. To validate this impression, we analyzed the implicit time of the 2nd oscillatory potentials peak for each animal (Figure 5C). As expected, the implicit time decreased with increasing flash intensity for both mouse groups but the WT group showed smaller inter-individual variability at each flash intensity compared to the Syne-2/Nesprin-2 KO mice. In Figure 5D, plots of the variances of the 2nd peak implicit times were indeed consistently larger in the Syne-2/Nesprin-2 KO group for all flash intensities. Statistical analysis of WT and Syne-2/Nesprin-2 KO variances revealed a significant difference for flashes larger than –2.2 phot log cd.s/m^2^ (Levene’s test; * *p* < 0.05, ** *p* < 0. 005). Photopic flicker ERG amplitudes and phases of Syne-2/Nesprin-2 KO and WT mice were similar (data not shown).

Based on this results we tested the localization of several proteins in the inner retina (e.g., Bm3A, GCL marker; PCKα, bipolar cell marker; VGlutT1, marker PRC and bipolar cell terminals) and found no differences in the structure of the retina and the distribution of the proteins between WT and the Syne-2/Nesprin-2 KO mice (data not shown).

### 3.4. Syne-2/Nesprin-2 KO Mice Show Reduced Syne-2 Levels

**Figure 6:**
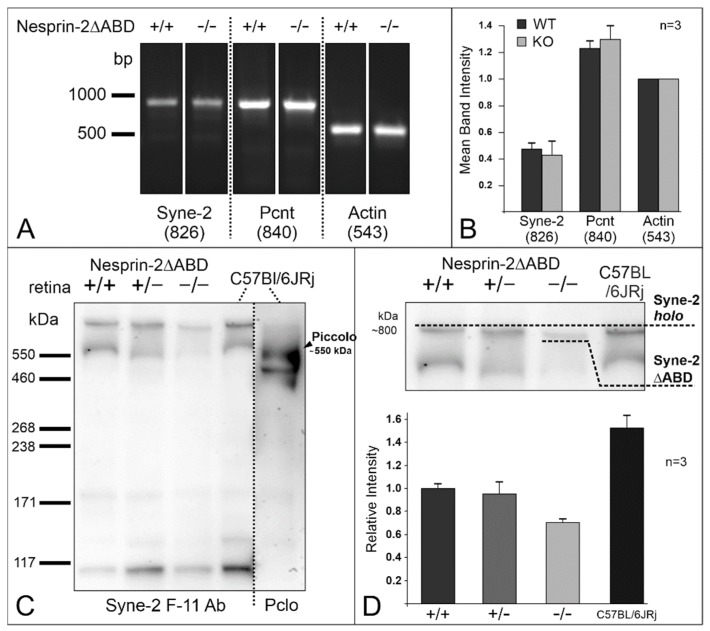
Expression of Syne-2 in Syne-2/Nesprin-2 KO retinae (age 2–4 months). (**A**) RT-PCR made with cDNA from Syne-2/Nesprin-2 KO and wild-type (WT) mouse retinae using specific primer sets for Syne-2, Pcnt, and β-actin (positive control). Syne-2 (aa residues 1921–2066) and Pcnt (aa residues 917–1169) are expressed in WT and KO retinae. The β-actin positive control demonstrates the approximate amount and the quality of the used cDNA. (**B**) Intensity measurements (*n* = 3) show an unchanged mRNA expression level of Syne-2 and Pcnt in Syne-2/Nesprin-2 KO and WT mouse retina with respect to the β-actin control. (**C**) Protein expression of Syne-2 in the mouse retina of C57BL/6JRj and in the different genotypes of the Syne-2/Nesprin-2 KO mouse line. Western blot analysis using the Syne-2^C-term^ antibody detect the full-length Syne-2 (~800 kDa), a splice-variant about 550 and 110 kDa in all Syne-2/Nesprin-2 KO phenotypes. An anti-Piccolo antibody was used for validating protein sizes detecting Piccolo (550 kDa). (**D**) Mean band intensity measurements of Western blot bands (stain-free imaging technology, BioRad) show a comparable expression level of full-length Syne-2 in all Syne-2/Nesprin-2 KO phenotypes, however, protein sizes varied. Retina extracts from the KO mouse show the presence of a Syne-2 protein with lower molecular weight than wild-type and heterozygous Syne-2/Nesprin-2 KO retina extracts implicating the expression of a smaller protein variant lacking the N-terminal part of Syne-2 (*n* = 3).

Based on the results of our previous study showing the presence of a functional interaction between Syne-2 and Pcnt [5], it was surprising to find no structural and only a mild functional phenotype in the Syne-2/Nesprin-2 KO retinae. We, therefore, performed a detailed expression analysis of Syne-2 and Pcnt in WT control and Syne-2/Nesprin-2 KO mice (Figure 6).

We amplified the region encoding the Pcnt interaction domain in Syne-2 (aa residues 1921–2066) and Pcnt (with primers amplifying all splice variants), using RNA from WT control and Syne-2/Nesprin-2 KO retinae as templates. Changes in expression levels were determined after normalization against the β-actin control (Figure 6A,B). The mRNA expression levels of the Pcnt interaction site in Syne-2 and of Pcnt were unaffected in the Syne-2/Nesprin-2 KO retinae compared to WT controls (Figure 6A,B).

Further Western blots analysis demonstrated the presence of ~550 and ~110 kDa Syne-2 splice variants in all Syne-2/Nesprin-2 KO and WT retina extracts. Full-length Syne-2 (~796 kDa) was detected in WT and heterozygous mice (Figure 6C,D). The largest Syne-2 protein identified in the homozygous Syne-2/Nesprin-2 KO retina extracts showed a slightly lower molecular weight compared to WT controls (Figure 6C,D); the relative protein levels were normalized to total protein using “stain-free technology.” In summary, our Western blot data suggest that the Syne-2/Nesprin-2 KO retina shows a slightly reduced Syne-2 protein level and a lower molecular weight of full-length Syne-2 compared to the WT control but there was no impairment of the Pcnt binding domain in Syne-2.

## 4. Discussion

Syne-2/Nesprin-2 belongs to the nuclear envelope spectrin superfamily. It is a known mediator of nuclear migration and positioning during retinal development in flies, zebrafish, and mice [1,2], and it has been shown to be involved in ciliogenesis [3,4]. We have previously demonstrated a potential functional interaction between Syne-2/Nesprin-2 and Pcnt, a protein of the pericentriolar material and the basal body complex [5,10]. The subcellular localization of Syne-2/Nesprin-2 and Pcnt in the murine retina partially overlapped with the basal body complex of the connecting cilium, and the deletion of the Pcnt interacting domain from Syne-2/Nesprin-2 (Pcnt binding epitope of Syne-2: aa residues 1921–2066, Figure 2C) affected ciliogenesis and ciliary length control, and seems to play a role in centrosomal-mediated nuclear migration [5]. The aim of the present study was to further characterize the functional relevance of the Syne-2/Nesprin-2-Pcnt interaction for the structure and function of the retina, using a Syne-2/Nesprin-2 KO mouse model. As the published Syne-2 KO mouse with a deletion of the Syne-2 KASH domain and showing a retinal phenotype [3] was no longer available (personal information Dr. Min Han, University of Colorado), we decided to employ the Syne-2/Nesprin-2 KO mouse model Nesprin-2△ABD (Syne-2^tm1Ngl^, MGI), which lacks the actin-binding domain (ABD) at the N-terminus of Syne-2. Expression analyses of the Nesprin-2△ABD mouse strain with the C-terminal antibody showed reduced expression of various smaller splice variants. The complete absence of protein variants with a molecular weight larger than 400 kDa (full-length protein 796 kDa) [12] and thus the lack of the complete N-terminal protein made the mouse model very interesting for us. We assumed that this deletion will also affect the structure of the remaining protein and therefore the Pcnt/Syne-2 binding site near the N-terminus of Syne-2 (Figure 2).

### 4.1. A Hypomorphic Mutation of Murine Syne-2 does not Affect Retina Structure

Syne-2/Nesprin-2 KO mice were generated by deleting three CH1 domain (calponin homology domain 1)-encoding exons (exons 3, 4, and 5), which, together with the CH2 domain, form a functional ABD at the N-terminus of Syne-2 [12]. To detect the variety of the splice forms of Syne-2, we used for expression analyses two monoclonal antibodies (antigen region at the N- or C-terminal region, Figure 1F). Here we were able to confirm weaker staining of Syne-2 in the Syne-2/Nesprin-2 KO photoreceptors but no difference in the localization of Syne-2 and its interaction partner Pcnt (Figure 1). The lack of any structural phenotype in the photoreceptors of Syne-2/Nesprin-2 KO mice (e.g., in the connecting cilium or in the thickness of the ONL, INL, or GCL) differs from the observations made in zebrafish, where structural defects are also associated with the ONL, INL, and GCL [3,20]. In addition, the lack of nuclear mislocalization caused by a disturbed migration of photoreceptors shown in the Syne-2 deleted KASH domain mouse cannot be explained here [3].

### 4.2. Nesprin-2△ABD KO Mice Show Altered Inner Retinal Signal Processing

To investigate putative functional defects in the retina of Syne-2/Nesprin-2 KO mice, we performed full-field ERG measurements on WT control and homozygous Syne-2/Nesprin-2 KO mice. The ERG results did not indicate a role of the Syne-2 N-terminus in photoreceptor transduction, because the a-waves were similar in both genotypes (Figure 4). Also signal transmission from photoreceptors to bipolar cells was inconspicuous, as the b-wave (reflecting bipolar cell activity [16]) was not affected in the Syne-2/Nesprin-2 KO mice. Interestingly, however, the scotopic oscillatory potentials showed a significantly larger interindividual variability for peak times in the Syne-2/Nesprin-2 KO mice (Figure 4). Currently, the mechanisms and contributors to the mouse oscillatory potentials are still unclear. It is agreed at least that they arise from complex activations of various inner retinal cells [16,17,21]. The changes in the oscillatory potentials in the Syne-2/Nesprin-2 KO mice are not easy to explain, given that the temporal change was neither consistently faster nor slower. Possibly, the Syne-2 protein plays a role in synchronizing inner retinal signal processes without affecting the major outer signal pathways.

### 4.3. Expression Analysis Revealed an Alternative Translational Start Site of Syne-2

To explain the absence of a strong structural phenotype in Syne-2/Nesprin-2 KO mice, we performed retinal expression analysis of Syne-2 on mRNA and protein level, which only showed slight changes (Figure 6). Only the protein level showed a slightly weaker expression of Syne-2 variants containing the Pcnt binding site in photoreceptor cells (Figure 6). Here, we identified, for the first time, a protein variant in the homozygous Syne-2/Nesprin-2 KO retina with a molecular weight larger than 400 kDa (~750 kDa) compared to other tissues [12]. The variant showed a lower molecular weight compared to the full-length WT protein (Figure 6C,D). This finding supports the hypothesis that a deletion of the CH1-domain in the Syne-2/Nesprin-2 KO mice might lead to a deletion of 215 amino acids from the N-terminus of Syne-2 by the usage of alternative translational start sites discussed by Lüke and colleagues [12]. This aberrant protein would lack the CH1 domain (encoded by exons 3–5), the linker region (exon 6), and at least 30 amino acids of the CH2 domain (Figure 2). Consequently, this altered structure would not affect the Pcnt interaction domain encoded by exons 39–41, which would explain why the interaction between Pcnt and Syne-2 is not severely affected in the Syne-2/Nesprin-2 KO mouse strain. Thus, the lack of a clear retinal phenotype in this mouse model did not provide any functional information on the role of Pcnt and Syne-2 interaction in the mouse retina.

## 5. Conclusions

Based on the results of our study, we could not find a structural retinal phenotype in Syne-2/Nesprin-2 KO mice at all because the N-terminal mutation leads only to an alternative transcription start of the protein Syne-2 of which only about 200 aa are affected at the N-terminus (Figure 2). The Pcnt interaction site is still expressed in the Syne-2/Nesprin-2 KO. Thus, this mouse model is not suitable to study the interaction of Syne-2 and Pcnt in vivo. To investigate an effect of the identified interaction between Pcnt and Syne-2, it would be necessary to generate a Syne-2/Nesprin-2 KO mouse with the stop mutation C-terminal to the Pcnt interaction site (exons 39–41) in the Syne-2/Nesprin-2 gene as shown in Falk et al. (Figure 2C) in the cell culture. Nevertheless, deletion of the actin-binding site in the Syne-2/Nesprin-2 KO mouse revealed high variability in scotopic oscillatory potentials, assuming a function of Syne-2 in synchronizing inner retinal processing, a finding that has to be studied in further electrophysiological experiments.
